# Relationships between Late-Life Blood Pressure and Cerebral Microinfarcts in Octogenarians: An Observational Autopsy Study

**DOI:** 10.3390/jcm12186080

**Published:** 2023-09-20

**Authors:** Mo-Kyung Sin, Yan Cheng, Jeffrey M. Roseman, Edward Zamrini, Ali Ahmed

**Affiliations:** 1 College of Nursing, Seattle University, Seattle, WA 98122, USA; 2 Biomedical Informatics Center and School of Medicine & Health Sciences, George Washington University, Washington, DC 20052, USA; yan_cheng@gwu.edu (Y.C.); ali.ahmed@va.gov (A.A.); 3 Department of Epidemiology, University of Alabama at Birmingham, Birmingham, AL 35233, USA; bush@uab.edu; 4 Irvine Clinical Research, Irvine, CA 92614, USA; 5 VA Medical Center, Washington, DC 20242, USA; 6 School of Medicine, Georgetown University, Washington, DC 20057, USA

**Keywords:** blood pressure, late life, microinfarcts, octogenarians

## Abstract

Mid-life high blood pressure (BP) is a risk factor for cerebral microinfarcts. Less is known about the relationship between late-life BP and cerebral microinfarcts, the examination of which is the objective of the current study. This case–control study analyzed data from 551 participants (94.6% aged ≥80 years; 58.6% women) in the Adult Changes in Thought (ACT) study who had autopsy data on microinfarcts and four values of systolic and diastolic blood pressure (SBP and DBP) before death. Using the average of four values, SBP was categorized using 10 mmHg intervals; a trend was defined as a ≥10 mmHg rise or fall from the first to fourth values (average gap of 6.5 years). Multivariable-adjusted regression models were used to examine the associations of BP and microinfarcts, adjusting for age, sex, last BP-to-death time, APOE genotype, and antihypertensive medication use. Microinfarcts were present in 274 (49.7%) participants; there were multiple in 51.8% of the participants, and they were located in cortical areas in 40.5%, subcortical areas in 29.6%, and both areas in 29.9% of the participants. All SBP categories (reference of 100–119 mmHg) and both SBP trends were associated with higher odds of both the presence and number of microinfarcts. The magnitude of these associations was numerically greater for subcortical than cortical microinfarcts. Similar associations were observed with DBP. These hypothesis-generating findings provide new information about the overall relationship between BP and cerebral microinfarcts in octogenarians.

## 1. Introduction

Cerebral microinfarcts, small (<5 mm) ischemic lesions, are a prevalent vasculopathy in older adults [1,2,3]. Hypertension, also highly prevalent in older adults, has detrimental effects on changes in the cerebral vascular structure and function, but less is known about the influence of hypertension on ischemia [4]. Even less is known about the temporal relationship between late-life blood pressure (BP) and microinfarcts, particularly among octogenarians, one of the fastest-growing segments of the US population. In one autopsy study of 297 older adults (mean age of 87.2 ± 5.3 years), 15.8% had chronic microinfarcts, of which 40.4% were subcortical. Mid-life baseline BP had no association with cerebral microinfarcts, but a declining BP was associated with subcortical microinfarcts [5]. In another autopsy study of 250 older participants in the Adult Changes in Thought (ACT) study, there was an association between late-life BP and cerebral microinfarcts, but this association was not observed in those (102) who were older than 80 years [6]. The objective of this study was to examine the BP associations in late life in a considerably larger and older sample of participants in the ACT study, almost all of whom died at over age 80 and half of whom died at over age 90. All subjects were selected to have four measures of BP over an approximately 6-year period, about one measure every 2 years, closest to death. We examined autopsy data from 551 ACT participants to determine (1) the prevalence of cerebral microinfarcts, (2) their burden (number), (3) regional distribution in cortical (frontal, temporal, parietal, and occipital) and subcortical (caudate nucleus, putamen, internal capsule, and thalamus) locations, and (4) their association with late-life BP (categories based on the mean of four values, trend based on the difference between values one and four, and variability based on standard deviation of the mean of the changes in four BP values from one to four).

## 2. Materials and Methods

### 2.1. Data and Participants 

We used data from the ACT study, an ongoing population-based cohort study of incident dementia, the details of which have been previously described [7,8]. Briefly, a random sample of 2581 adults 65 years and older, free of dementia at baseline and who were members of Kaiser Permanente Washington (previously Group Health) in the Seattle region, were enrolled in 1994–1996 and followed for 28 years. ACT subsequently enrolled a cohort of 811 patients in 2000–2003 with the same methods, which was continued to replace attrition from dementia, dropout, and death, ensuring a consistent cohort of ≥2000 at risk for dementia. Total enrollment over the course of the study is >5000 with a total of 850 autopsies available at the time of this analysis. 

**Figure 1:**
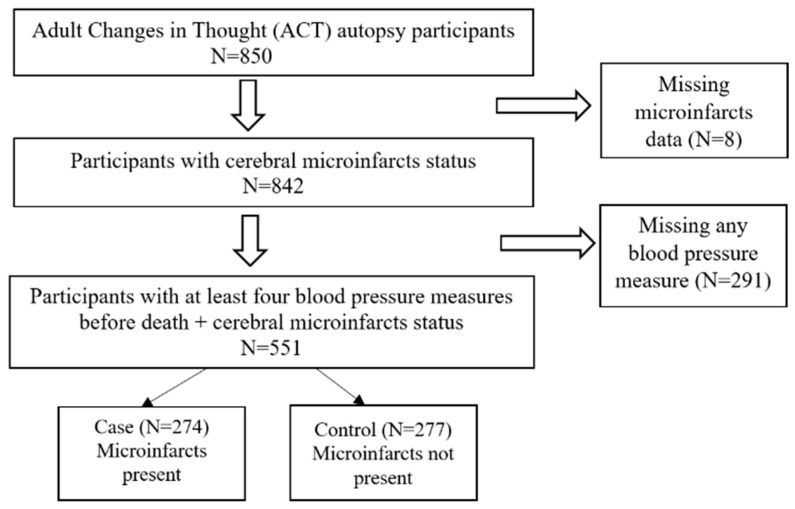
Flow Chart.

The current study was based on 551 participants who had complete data on the presence, count, and locations of microinfarcts, as well as four measures of BP before death (Figure 1). Exclusion criteria were those with any missing values on the microinfarcts and the last four BP measurements before death. The study protocols were approved by Kaiser Permanente Institutional Review Boards. All participants in the ACT study reviewed and signed a standard consent form before they joined the study and completed any study activities. During enrollment, participants were asked whether they would give permission for a post-mortem examination of their brain. 

### 2.2. Classification of Cases and Controls

Cases were defined by the presence of one or more microinfarcts in any of the bilateral sections of cortical (frontal lobe, temporal lobe, parietal lobe, and occipital lobe) and subcortical (caudate nucleus, putamen, internal capsule, and thalamus) regions of the brain. All ACT brains underwent Hematoxylin–Eosin and Luxol Fast Blue staining for every sampled region as part of the standard diagnostic neurohistologic workup [9]. All brains were evaluated for chronic (more than 24 h old) microinfarcts, as these lesions are more readily associated with factors relevant during life and cognitive impairment (vs. acute microinfarcts reflecting peri-mortem events) [10]. Extensive neuropathological examinations were performed following the current diagnostic criteria, including the National Institute on Aging-Alzheimer’s Association (NIA-AA) consensus guidelines, the details of which have been previously described [8,11]. Briefly, neuropathological examinations were performed by board-certified neuropathologists blinded to the clinical diagnosis, status of risk factors, and BP readings. Each case was assessed by one board-certified neuropathologist, but challenging cases were discussed in consensus with 1–2 neuropathologists. Based on the judgments of the neuropathologists, microinfarcts were categorized as one and more than one. The observation of 1 or 2 microinfarcts suggests the likely presence of hundreds of microinfarcts in the sample region [1]. Those without any microinfarct were considered controls. 

### 2.3. Study Exposure

BPs were measured at the time of enrollment and at each biennial follow-up by a trained nurse or a technician using standardized techniques with patients in a seated position after resting for at least 5 min. The accuracy of BP monitors was assessed before each use by checking whether the needle was resting exactly at the middle of zero when the air bladder was deflated. BP devices were calibrated yearly by the University of Washington Scientific Instruments group. SBP was defined as the reading at the first Korotkoff sound and DBP (DBP) as the reading at the disappearance of the Korotkoff sound. BPs were measured two times at intervals of 5 min, and the mean was calculated. For the purpose of the current study, we defined late-life BP as the last four consecutive BP measurements from ACT visits closest to death in this study. It has been suggested that a minimum of a 5-year time period is required for the progressive development of vascular impairment to occur [12].

### 2.4. Covariates

Trained research personnel obtained a detailed medical history and medication use at baseline and each biennial visit using a standardized questionnaire. Data on the use of antihypertensive medications were collected from participants’ self-reports in the four consecutive visits. Antihypertensive medication use was assessed using the question “Are you taking prescribed medication for BP now?” and was categorized as “never used”, “current user”, and “past user” in four consecutive data points. In this study, if a patient was a “current user” during the last four visits, then the patient would be classified as ever used antihypertensive medication. Other covariates included age at death, sex, time gap between last BP and death date (years), and APOE [13,14,15]. APOE genotyping was performed using two independent procedures: Taqman assay and Sanger sequencing. Their matching genotype was then reported. APOE e4 status is rated as 0 = absence of the e4 allele or 1 = presence of 1 or 2 e4 alleles.

### 2.5. Statistical Analysis

Since BP was the main exposure and microinfarcts were the outcome, we selected the study population with complete data on both the exposure and outcome. We did not exclude any participants if they had a missing value in any other covariates. Categorical variables with missing values were categorized as “unknown”. 

Characteristics of participants with and without microinfarcts were compared using chi-square and Student’s t-tests. For primary analyses, we categorized the mean of the last 4 SBPs into 100–119 (reference), 120–129, 130–139, 140–149, 150–159, 160–169, or ≥170 mmHg. We defined a “rise” or “fall” in SBP from the first to the last (fourth) using a cutoff of 10 mmHg. To define variability, we calculated the differences in BP between the first and the second, the second and the third, and the third and the fourth measurements for each individual. We then calculated the mean and SD of these 3 differences for each individual. We then calculated the mean and SD of these measures across all subjects. Lastly, we compared the SD of each individual with the SD across all individuals. If the SD was less than the mean of the SDs, it was classified as “least variable”; if it was greater than the mean of the SDs but less than one SD of the mean greater than the mean of the SDs, it was classified “moderately variable”; and if it was greater than one SD from the mean of the SDs, it was classified “extremely variable”. We then used separate multivariable logistic regression models for SBP categories for the presence and location of microinfarcts and ordinal multivariable logistic regression models for changes in and the variability of BP. The models for the presence, number, and regional distribution of microinfarcts were adjusted for age at death, sex, antihypertensive medication use, APOE genotype, and duration between the last BP and death. The models for BP change and variability were also adjusted for the time between the first and last BP measures.

## 3. Results

### 3.1. Participant Characteristics

Microinfarcts were present in 274 (49.7%) participants. A total of 132 (48.2%) had one; 142 (51.8%) had multiple; for 111 (40.5%), they were cortical; for 81 (29.6%), they were subcortical; and for 82 (29.9%), they were both. We did not find any significant differences in the sub-analysis between the 551 participants and the 291 excluded from this study for missing values. Among the 291 non-participants, 148 (50.9%) had no microinfarcts and 143 (49.1%) had microinfarcts. Among the participants, the mean SBP was 137 (±16) mmHg, and the mean DBP was 71 (±8) mmHg. Seventy-one (12.9%) had consistently high SBP measures (≥140 mmHg for all four measures), and two (0.4%) had consistently high DBP (≥90 mmHg for all four measures). The mean time difference was 3.1 (SD 2.7) years for the duration between the last BP and death and 6.5 (SD 1.4) years for the duration between the first and last BP measures of the last four consecutive BP measurements. Compared to participants without microinfarcts, those with microinfarcts were older, but there were no between-group sex or race differences (Table 1). Those with microinfarcts also had a higher prevalence of dementia and hypertension. 

### 3.2. Association of Late-Life BP with Presence and Number of Microinfarcts

The mean last SBP was higher in the group with microinfarcts (135.9 mmHg vs. 132.3 mmHg in the non-microinfarct group), which was almost statistically significant (*p* = 0.05, Table 1), and the mean last DBP was significantly higher in the microinfarct group (72.0 mmHg vs. 69.7 mmHg, *p* = 0.015; Table 1). Most of the final BP measures were taken within 5 years of death (77.5%) and 46.8% within the final 2 years. More participants with dementia had no BP measures within 5 years prior to death (38.7%) compared to those without (5.6%).

The mean of the last four SBP measurements was higher in the group with microinfarcts (139.7 mmHg) than in the non-microinfarct group (134.6 mmHg), which was statistically significant (*p* < 0.011, Table 2). Similarly, the mean of the last four DBP measurements was also significantly higher in the microinfarct group (72.3 mmHg vs. 70.5 mmHg, *p* = 0.01; Table 2). The mean of the last four PP measurements was higher in the group with microinfarcts (67.4 mmHg) than in the non-microinfarct group (64.1 mmHg), which was statistically significant (*p* < 0.011, Table 2).

Compared with participants with a mean SBP of 100–119 mmHg, the odds ratios (95% CI) for the presence of cerebral microinfarcts associated with an SBP of 120–129, 130–139, 140–149, 150–159, and 160–169 mmHg were 2.22 (1.18–4.18), 2.71 (1.48–4.98), 2.09 (1.10–3.98), 2.87 (1.40–5.92), and 2.69 (1.13–6.39), respectively (Table 3). After covariable adjustment, compared with participants with a mean SBP of 100–119 mmHg, the odds ratios (95% CI) for the number of cerebral microinfarcts associated with an SBP of 120–129, 130–139, 140–149, 150–159, and 160–169 mmHg were 2.01 (1.10–3.69), 2.68 (1.50–4.80), 2.11 (1.14–3.90), 2.55 (1.28–5.04), and 2.66 (1.18–5.98), respectively (Table 3). Although only 18 (3.3%) participants had an SBP of 170 mmHg or higher, the odds ratio for that group showed nearly four times higher odds of having any microinfarcts as well as of having more than one microinfarct, both of which were significant (Table 3). Odds ratios (95% CI) for the presence and number of microinfarcts associated with an SBP ≥170 were 5.10 (1.59–16.32) and 4.68 (1.71–12.80), respectively (Table 3). When SBP was categorized as a trend, the odds ratios (95% CI) for the presence of cerebral microinfarcts associated with a 10 mmHg rise and fall in SBP were 1.99 (1.27–3.11) and 2.05 (1.35–3.12), and the respective odds ratios (95% CI) for the number of microinfarcts were 1.99 (1.31–3.02) and 1.91 (1.29–2.84), respectively (Table 3). SBP variability had no significant association with microinfarcts (Table 3). 

Compared with participants with a mean DBP of 60–64 mmHg, the odds ratios (95% CI) for the presence of cerebral microinfarcts associated with a DBP of 40–59, 65–69, 70–74, 75–79, 80–84, and ≥85 mm Hg were 1.44 (0.63–3.26), 2.11 (1.15–3.87), 2.21 (1.21–4.04), 2.82 (1.46–5.48), 2.19 (1.05–4.58), and 3.07 (1.21–7.79), respectively (Table 3). DBP deciles had similar associations with the number of cerebral microinfarcts (Table 3). The DBP trend had no significant association with microinfarcts. Compared with participants with the least DBP variability, the odds ratios (95% CI) for the presence of cerebral microinfarcts associated with moderate and extreme DBP variability were 1.69 (1.13–2.54) and 1.43 (0.87–2.37), respectively, and the odds ratios (95% CI) for the number of microinfarcts were 1.71 (1.18–2.49) and 1.29 (0.81–2.05), respectively (Table 3).

### 3.3. Association of Late-Life BP with Cortical and Subcortical Microinfarcts

Compared with participants with a mean SBP of 100–119 mmHg, the odds ratios (95% CI) for the presence of cortical microinfarcts associated with an SBP of 120–129, 130–139, 140–149, 150–159, 160–169, and ≥170 mm Hg were 1.46 (0.75–2.85), 1.95 (1.03–3.70), 1.28 (0.65–2.54), 2.07 (0.98–4.37), 1.62 (0.66–3.99), and 1.99 (0.67–5.94), respectively (Table 4). Compared with participants with a mean DBP of 60–64 mmHg, the odds ratios (95% CI) for the presence of cerebral microinfarcts associated with a DBP of 40–59, 65–69, 70–74, 75–79, 80–84, and ≥85 mmHg were 1.27 (0.54–2.98), 1.34 (0.71–2.51), 1.13 (0.60–2.13), 1.50 (0.76–2.95), 1.49 (0.70–3.17), and 2.77 (1.10–6.98), respectively (Table 4). Associations of SBP and DBP trends and variability with cortical microinfarcts are displayed in Table 4.

Compared with participants with a mean SBP of 100–119 mmHg, the odds ratios (95% CI) for the presence of subcortical microinfarcts associated with an SBP of 120–129, 130–139, 140–149, 150–159, 160–169, and ≥170 mmHg were 1.70 (0.80–3.62), 2.06 (1.00–4.23), 2.51 (1.19–5.32), 2.01 (0.87–4.63), 3.10 (1.20–8.00), and 8.80 (2.71–28.53), respectively (Table 4). Compared with participants with a mean DBP of 60–64 mmHg, the odds ratios (95% CI) for the presence of cerebral microinfarcts associated with a DBP of 40–59, 65–69, 70–74, 75–79, 80–84, and ≥85 mmHg were 2.93 (1.04–8.26), 3.52 (1.54–8.08), 4.77 (2.11–10.82), 3.92 (1.64–9.36), 3.85 (1.51–9.80), and 3.72 (1.24–11.17), respectively (Table 4). Associations of SBP and DBP trends and variability with subcortical microinfarcts are displayed in Table 4.

### 3.4. The Associations of the Covariates with Microinfarcts

Compared with participants aged <80 years, the odds ratios (95% CI) for the presence of cerebral microinfarcts associated with age 80–89 and >−90 years were 3.33 (1.28–8.64) and 3.62 (1.40–9.35), respectively (Table 5). Compared with participants aged <80 years, the odds ratios (95% CI) for the number of cerebral microinfarcts associated with age 80–89 and >−90 years were 3.67 (1.41–9.58) and 3.81 (1.47–9.88), respectively (Table 5). Cerebral microinfarcts did not have significant associations with other participant characteristics (Table 5). 

## 4. Discussion

The findings from our study demonstrated that a higher SBP in the last decade of life had a strong association with autopsy-documented cerebral microinfarcts in octogenarians, but this association did not carry a dose–response relationship. We also demonstrated that a drop or rise, but not the variability, in SBP during this period also had similar associations with the presence and number of microinfarcts, which also lacked a dose–response relationship. Furthermore, we observed that the association of high SBP with microinfarcts was more pronounced for subcortical than for cortical microinfarcts. A rise or drop in SBP was similarly associated with both subcortical and cortical microinfarcts. Finally, DBP had similar associations, albeit modest, with microinfarcts, which was somewhat attenuated when adjusted for SBP. Considering that hypertension is a powerful risk factor for cerebrovascular pathology, leading to stroke and dementia [16,17,18,19], new information about the relationship between BP in the last decade of life and cerebral microinfarcts in octogenarians from the current study may provide insights into the role of cerebral microinfarcts in the pathway between BP and cognitive decline and dementia. 

There were only a few millimeters of mercury differences in average BP between those with and without microinfarcts. For example, based on Table 1, the mean last SBP was only 3.6 mmHg higher in the group with microinfarcts (135.9 mmHg vs. 132.3 mmHg in the non-microinfarct group), although this result was almost statistically significant (*p* = 0.05, Table 1). In our analyses, we did not compare the association with the continuous variable, the mean of the last four SBPs. We categorized the mean of four SBP values by deciles of SBP and observed that higher deciles of the four SBP values were associated with a higher risk of microinfarcts, but there was no dose-dependent incremental association with microinfarcts until SBP reached ≥170 mmHg. We also found that a rise or drop in SBP ≥10 mmHg is also associated with higher odds of microinfarcts.

Persistently elevated BP as in patients with hypertension is a risk factor for intracranial atherosclerosis, vascular remodeling, and collagenosis [17,20,21,22]. These macrovascular changes result in stiffened arteries, impaired blood flow, and then thrombogenesis, leading to microscopic ischemic or hemorrhagic lesions and ultimately causing microinfarcts [17,20]. Although less is known about the exact natural history of the pathogenesis of high-BP-mediated microinfarcts, it is often associated with mid-life high BP, suggesting that prolonged exposure to high BP may be necessary. While the association between mid-life BP and dementia is better known [23,24,25], less is known about the possible mediating role of cerebral microinfarcts when its risk factors (e.g., age and genetics (APOE e4)) are controlled. The findings from the Atherosclerosis Risk in Communities study (n = 4761) support the mediating role of microinfarcts in the pathway of BP to dementia [25,26,27]. Future studies can examine the pathway from late-life BP to microinfarcts to dementia.

Prior studies have examined the association between BP and cerebral microinfarcts, most of which are limited to small sample sizes and few octogenarians [5,6]. To the best of our knowledge, this is the largest autopsy study of octogenarians, which provides new information regarding the complex relationship of BP with the presence and number of microinfarcts in cortical and subcortical areas of the brain. Several observations from our study suggest that microinfarcts in octogenarians may have a different relationship with late-life BP than with mid-life BP. High BP has been shown to have a graded or incremental association with macrovascular cardiovascular complications [28]. However, we observed no such association between late-life high BP and cerebral microinfarcts in the octogenarian population in our study. This is consistent with the relationship between high SBP and intracranial atherosclerotic stenosis, which has lacked a dose–response relationship until above 170 mmHg [29]. A low DBP has been shown to be associated with a higher risk of both macrovascular and microvascular complications including microinfarcts [5]. In contrast, we observed that high DBP was associated with a higher risk of microinfarcts, and, importantly, this association was independent of SBP. The association between DBP and macrovascular complications has been shown to be attenuated when adjusted for SBP [30]. We also observed that both a decline and rise in BP during the last decade of life, but not variation in BP, was associated with a higher risk of microinfarcts. 

Others have reported a significant association between day-to-day BP variability (measured as ((SD/mean BP) × 100)) and dementia [31]. A systematic review and meta-analysis also reported a significant association between BP variability and cerebral small-vessel diseases [32]. The lack of association of SBP variability with microinfarcts despite evidence of increased associations with a trend of either an increase or decrease in SBP by 10 mmHg or more is intriguing in our study. One possible explanation is that the BP change dynamics captured by our definition of variability are different from those of an upward or downward trend in BP. The association of DBP variability with microinfarcts is also intriguing considering that trends in DBP had no significant associations. The fact that the association was only observed for moderate variability but not with extreme variability suggests that there likely was no dose–response relationship or biological plausibility and that this may be a chance observation. Although it is beyond the scope of the current study to examine the underlying reasons for these hypothesis-generating observations, they provide insights into the potentially complex relationship between late-life BP and cerebral microinfarcts in octogenarians.

Hypertension leads to structural changes in vascular walls in the deep brain regions including white matter [33,34], leading to subcortical microinfarcts. While the mechanisms of microinfarcts are heterogeneous, we looked at only BP in this study. If these findings can be replicated, future studies will need to develop and test novel strategies for BP control in octogenarians that avoid a high BP but also avoid a rise or fall in BP ≥10 mmHg in the last decade of life and will also need to determine if the successful use of those strategies can reduce the risk of microinfarcts. This is important considering that octogenarians are one of the fastest growing segments of the US population who are also at a very high risk for dementia. 

### Limitations

Several limitations of our study need to be acknowledged. As in any observational study, there is possible bias due to residual or unmeasured confounders, including other risk factors for microinfarcts such as cerebral small vessel disease (e.g., cerebral amyloid angiopathy and arteriolosclerosis), microemboli, and hypoperfusion [35]. The analysis was based on limited regions of the brain (four regions for both cortical and subcortical), which may underestimate the total microinfarct burden in other regions. In addition, a standard autopsy of the brain takes only a few samples (usually ≤20) for processing into paraffin sections (approximately 4–6 µm thick) for histopathological analysis, meaning less than 0.01% of the entire brain is sampled [10]. This indicates that many cerebral microinfarcts throughout the brain are not detected [35]. The autopsy cohort is a voluntary group, and, thus, the study outcomes may not be comparable with random people. Because BP was measured at specific time points during 2-year follow-up visits, the variability described in our study may not correctly reflect within-day or day-to-day variability. Furthermore, because BP was measured in the office setting (research clinic), we were not able to account for white coat hypertension, which may misclassify patients with normal BP as having hypertension [36]. Although this has the potential to attenuate associations between BP and microinfarcts, this is likely a lesser concern as BP was categorized as deciles in our study. Other limitations include the lack of mid-life BP data, the use of only the last four measures of BP, and limited information about the duration and frequency of medication, as participants self-reported antihypertensive medication use. The findings from this study are based on an over 90% non-Hispanic white urban-dwelling population enrolled in one of the largest managed care systems who volunteered to participate in a research study and may not be generalizable to other populations.

## 5. Conclusions

The findings of the current study demonstrate that both a high BP and a rise or drop in BP during the last decade of life have strong associations with cerebral microinfarcts in octogenarians. This new information provides insights into the complex relationship between BP and microinfarcts during the last decade of life of octogenarians who are one of the fastest growing segments of the population with one of the highest risks of dementia. Future studies need to develop and test optimal BP management strategies to minimize the risk of microinfarcts in octogenarians.

## Figures and Tables

**Table 1:** Participant Characteristics by Microinfarcts Status (N = 551).

Variables, n (%) or Mean (SD)	Microinfarcts Not Present (N = 277)	Microinfarcts Present (N = 274)	*p*-Value
Age categories			<0.001
<80 years	24 (8.7%)	6 (2.2%)	
80–89 years	110 (39.7%)	101 (36.9%)	
≥90 years	143 (51.6%)	167 (60.9%)	
Sex			0.559
Male	118 (42.6%)	110 (40.1%)	
Female	159 (57.4%)	164 (59.9%)	
Race White	256 (92.4%)	259 (94.5%)	0.581
Education ≤high school	126 (45.5%)	137 (50.0%)	0.454
Marital status Never married/separated/divorced/widowed	178 (64.3%)	186 (67.9%)	0.040
Ever smoker	151 (54.5%)	149 (54.4%)	0.603
Stroke	23 (8.3%)	37 (13.5%)	0.050
Heart disease	84 (30.3%)	95 (34.7%)	0.276
Diabetes	43 (15.5%)	45 (16.4%)	0.773
Dementia	109 (39.4%)	139 (50.7%)	0.002
APOE e4 carrier			0.169
No	199 (71.8%)	199 (72.6%)	
Yes	75 (27.1%)	66 (24.1%)	
Unknown	3 (1.1%)	9 (3.3%)	
BRAAK *			0.268
Stage 0	7 (2.5%)	2 (0.7%)	
Stage I–II	56 (20.2%)	47 (17.2%)	
Stage III–IV	111 (40.1%)	113 (41.2%)	
Stage V–VI	103 (37.2%)	112 (40.9%)	
Unknown	7 (2.5%)	2 (0.7%)	
Last systolic blood pressure (mmHg)	132.3 (21.6)	135.9 (21.6)	0.051
Last diastolic blood pressure (mmHg)	69.7 (10.7)	72.0 (12.1)	0.015
Ever hypertension (self-report)	188 (67.9%)	201 (73.4%)	0.158
Ever used anti-hypertensive medications	106 (38.3%)	117 (42.7%)	0.289
Ever used anti-lipid medications	112 (40.4%)	126 (46.0%)	0.188
Time of last vital sign before death			
Within 1 year	63 (22.7%)	49 (17.9%)	0.075
1–<2 years	82 (29.6%)	64 (23.4%)	
2–<5 years	80 (28.9%)	100 (36.5%)	
5+ years	52 (18.8%)	61 (22.3%)	
Vital sign not available	63 (22.7%)	49 (17.9%)	

* BRAAK stage for neurofibrillary tangles is categorized as 0 = none (normal), 1 = I (very mild), 2 = II (mild), 3 = III (moderate), 4 = IV (moderately severe), 5 = V (severe), 6 = VI (very severe).

**Table 2:** Participants with both MIFs and last four BPs (N = 551).

Late-Life BP	Microinfarcts			Microinfarct Number				Microinfarct Cortical			Microinfarct Subcortical		
	Not Present	Present	*p*	None	One	Multiple	*p*	Not Present	Present	*p*	Not Present	Present	*p*
N	277	274		277	132	142		358	193		388	163	
Mean SBP	134.6 (16.4)	139.7 (15.8)	0.0002	134.6 (16.4)	138.8 (15.7)	140.5 (15.8)	0.0008	136.0 (16.4)	139.2 (15.8)	0.0248	135.4 (15.9)	141.5 (16.4)	<0.0001
Mean DBP	70.5 (8.2)	72.3 (8.2)	0.0104	70.5 (8.2)	72.5 (8.1)	72.1 (8.3)	0.0345	71.0 (8.0)	72.1 (8.7)	0.1071	70.9 (8.3)	72.5 (8.1)	0.0390
Mean PP	64.1 (13.7)	67.4 (13.5)	0.0046	64.1 (13.7)	66.3 (13.1)	68.5 (13.8)	0.0078	65.0 (14.0)	67.1 (13.0))	0.0914	64.4 (13.0)	69.0 (14.7)	0.0003

**Table 3:** Association of late-life systolic (top panel) and diastolic (bottom panel) blood pressure with presence (left panel) and number (right panel) of cerebral microinfarcts.

		Presence of Microinfarcts		Number of Microinfarcts	
		Adjusted * Odds Ratio (95% CI)	*p* Values	Adjusted * Odds Ratio (95% CI)	*p* Values
Systolic blood pressure (mmHg)	SBP categories ^1^				
	100–119 (n = 75)	1.00		1.00	
	120–129 (n = 113)	2.22 (1.18–4.18)	0.013	2.01 (1.10–3.69)	0.024
	130–139 (n = 143)	2.71 (1.48–4.98)	0.001	2.68 (1.50–4.80)	0.001
	140–149 (n = 106)	2.09 (1.10–3.98)	0.024	2.11 (1.14–3.90)	0.018
	150–159 (n = 63)	2.87 (1.40–5.92)	0.004	2.55 (1.28–5.04)	0.007
	160–169 (n = 33)	2.69 (1.13–6.39)	0.025	2.66 (1.18–5.98)	0.019
	170+ (n = 18)	5.10 (1.59–16.32)	0.006	4.68 (1.71–12.80)	0.003
	SBP trend ^2^				
	No change (n = 179)	1.00		1.00	
	Rise (n = 160)	1.99 (1.27–3.11)	0.003	1.99 (1.31–3.02)	0.001
	Fall (n = 212)	2.05 (1.35–3.12)	0.001	1.91 (1.29–2.84)	0.001
	SBP variability ^3^				
	None (n = 318)	1.00		1.00	
	Moderate (n = 145)	0.99 (0.66–1.49)	0.972	1.14 (0.78–1.67)	0.485
	Extreme (n = 88)	1.04 (0.64–1.70)	0.864	1.08 (0.69–1.70)	0.738
Diastolic blood pressure (mmHg)	DBP categories ^1^				
	60–64 (n = 73)	1.00		1.00	
	40–59 (n = 38)	1.44 (0.63–3.26)	0.385	1.66 (0.76–3.63)	0.206
	65–69 (n = 130)	2.11 (1.15–3.87)	0.016	2.09 (1.17–3.75)	0.013
	70–74 (n = 141)	2.21 (1.21–4.04)	0.010	2.41 (1.35–4.29)	0.003
	75–79 (n = 86)	2.82 (1.46–5.48)	0.002	2.41 (1.29–4.52)	0.006
	80–84 (n = 55)	2.19 (1.05–4.58)	0.037	2.06 (1.02–4.15)	0.044
	85+ (n = 28)	3.07 (1.21–7.79)	0.018	3.23 (1.38–7.58)	0.007
	DBP trend ^2^				
	No change (n = 154)	1.00		1.00	
	Rise (n = 193)	1.49 (0.96–2.31)	0.079	1.42 (0.94–2.14)	0.096
	Fall (n = 204)	1.20 (0.78–1.86)	0.408	1.22 (0.81–1.84)	0.337
	DBP variability ^3^				
	None (n = 315)	1.00		1.00	
	Moderate (n = 152)	1.69 (1.13–2.54)	0.011	1.71 (1.18–2.49)	0.005
	Extreme (n = 84)	1.43 (0.87–2.37)	0.158	1.29 (0.81–2.05)	0.282

* Adjusted for age at death, APOE, female, hypertension medication, time gap between last BP and death date (years). ^1^ Mean of the BPs across all 4 measures. ^2^ A rise was defined as BP ≥10 mmHg rise from the first to the last measures, and a fall was defined as ≥10 mmHg drop from the first to the last measures. ^3^ Standard deviation of the mean of the differences between subsequent measurement.

**Table 4:** Association of late-life systolic (top panel) and diastolic (bottom panel) blood pressure with presence (left panel) and number (right panel) of cerebral microinfarcts.

		Presence of Cortical MIF		Presence of Subcortical MIF	
		Adjusted * Odds Ratio (95% CI)	*p* Values	Adjusted * Odds Ratio (95% CI)	*p* Values
Systolic blood pressure (mmHg)	SBP categories ^1^				
	100–119 (n = 75)	1.00		1.00	
	120–129 (n = 113)	1.46 (0.75–2.85)	0.269	1.70 (0.80–3.62)	0.169
	130–139 (n = 143)	1.95 (1.03–3.70)	0.039	2.06 (1.00–4.23)	0.050
	140–149 (n = 106)	1.28 (0.65–2.54)	0.471	2.51 (1.19–5.32)	0.016
	150–159 (n = 63)	2.07 (0.98–4.37)	0.055	2.01 (0.87–4.63)	0.102
	160–169 (n = 33)	1.62 (0.66–3.99)	0.296	3.10 (1.20–8.00)	0.019
	170+ (n = 18)	1.99 (0.67–5.94)	0.216	8.80 (2.71–28.53)	< 0.001
	SBP trend ^2^				
	No change (n = 179)	1.00		1.00	
	Rise (n = 160)	1.72 (1.08–2.75)	0.022	2.29 (1.39–3.77)	0.001
	Fall (n = 212)	1.62 (1.04–2.51)	0.032	2.05 (1.27–3.29)	0.003
	SBP variability ^3^				
	None (n = 318)	1.00		1.00	
	Moderate (n = 145)	1.30 (0.85–1.97)	0.221	0.89 (0.57–1.38)	0.593
	Extreme (n = 88)	1.56 (0.95–2.57)	0.077	0.78 (0.46–1.34)	0.369
Diastolic blood pressure (mmHg)	DBP categories ^1^				
	60–64 (n = 73)	1.00		1.00	
	40–59 (n = 38)	1.27 (0.54–2.98)	0.580	2.93 (1.04–8.26)	0.043
	65–69 (n = 130)	1.34 (0.71–2.51)	0.365	3.52 (1.54–8.08)	0.003
	70–74 (n = 141)	1.13 (0.60–2.13)	0.680	4.77 (2.11–10.82)	< 0.001
	75–79 (n = 86)	1.50 (0.76–2.95)	0.243	3.92 (1.64–9.36)	0.002
	80–84 (n = 55)	1.49 (0.70–3.17)	0.305	3.85 (1.51–9.80)	0.005
	85+ (n = 28)	2.77 (1.10–6.98)	0.031	3.72 (1.24–11.17)	0.019
	DBP trend ^2^				
	No change (n = 154)	1.00		1.00	
	Rise (n = 193)	1.44 (0.91–2.28)	0.122	1.67 (1.03–2.69)	0.037
	Fall (n = 204)	1.29 (0.81–2.04)	0.286	1.14 (0.70–1.87)	0.591
	DBP variability ^3^				
	None (n = 315)	1.00		1.00	
	Moderate (n = 152)	1.23 (0.81–1.86)	0.324	1.95 (1.27–2.99)	0.002
	Extreme (n = 84)	1.19 (0.71–1.98)	0.513	1.38 (0.80–2.35)	0.246

* Adjusted for age at death, APOE, female, hypertension medication, time gap between last BP and death date (years). ^1^ Mean of the BPs across all 4 measures. ^2^ A rise was defined as BP rise of ≥10 mmHg from the first to the last measures, and a fall was defined as ≥10 mmHg drop from the first to the last measures. ^3^ Standard deviation of the mean of the differences between subsequent measurements.

**Table 5:** Other predictors of presence (left panel) and number (right panel) of cerebral microinfarcts.

	Presence of Microinfarcts		Number of Microinfarcts	
Covariates	Adjusted * Odds Ratio (95% CI)	*p* Values	Adjusted * Odds Ratio (95% CI)	*p* Values
Age 80–89 vs. <80 years	3.33 (1.28–8.64)	0.014	3.67 (1.41–9.58)	0.008
Age ≥ 90 vs. <80 years	3.62 (1.40–9.35)	0.008	3.81 (1.47–9.88)	0.006
Female	0.98 (0.68–1.40)	0.905	0.98 (0.70–1.38)	0.917
APOE e4 carrier Yes vs. No	0.84 (0.56–1.26)	0.389	0.95 (0.65–1.39)	0.789
APOE e4 carrier Unknown vs. No	2.83 (0.73–10.91)	0.132	1.32 (0.45–3.92)	0.614
Antihypertensive medication	1.17 (0.77–1.76)	0.463	1.15 (0.79–1.70)	0.467
Time gap between last BP and death day (years)	1.05 (0.98–1.13)	0.154	1.06 (0.99–1.13)	0.092

* Adjusted for SBP categories and all covariates listed in Table 3.

## Data Availability

The data presented in this study are available on request from the ACT. For more information you can visit: https://actagingstudy.org/ (accessed on 17 September 2023).
